# Inhibition of miR-101-3p prevents human aortic valve interstitial cell calcification through regulation of CDH11/SOX9 expression

**DOI:** 10.1186/s10020-023-00619-4

**Published:** 2023-02-21

**Authors:** Jianglei Chen, Yi Lin, Zhongjie Sun

**Affiliations:** 1 Department of Physiology, College of Medicine, University of Oklahoma Health Sciences Center, Oklahoma City, OK 73104 USA; 2 Department of Physiology, College of Medicine, UT Cardiovascular Institute, University of Tennessee Health Science Center, 956 Court Avenue, Memphis, TN 38163 USA

**Keywords:** Asporin, Osteocalcin, Osteopontin, Osteoblast, BMP2, Runx2, RNA-Seq, Anti-miR, Fetuin A, Differentiation

## Abstract

**Background:**

Calcific aortic valve disease (CAVD) is the second leading cause of adult heart diseases. The purpose of this study is to investigate whether miR-101-3p plays a role in the human aortic valve interstitial cells (HAVICs) calcification and the underlying mechanisms.

**Methods:**

Small RNA deep sequencing and qPCR analysis were used to determine changes in microRNA expression in calcified human aortic valves.

**Results:**

The data showed that miR-101-3p levels were increased in the calcified human aortic valves. Using cultured primary HAVICs, we demonstrated that the miR-101-3p mimic promoted calcification and upregulated the osteogenesis pathway, while anti-miR-101-3p inhibited osteogenic differentiation and prevented calcification in HAVICs treated with the osteogenic conditioned medium. Mechanistically, miR-101-3p directly targeted cadherin-11 (CDH11) and Sry-related high-mobility-group box 9 (SOX9), key factors in the regulation of chondrogenesis and osteogenesis. Both CDH11 and SOX9 expressions were downregulated in the calcified human HAVICs. Inhibition of miR-101-3p restored expression of CDH11, SOX9 and ASPN and prevented osteogenesis in HAVICs under the calcific condition.

**Conclusion:**

miR-101-3p plays an important role in HAVIC calcification through regulation of CDH11/SOX9 expression. The finding is important as it reveals that miR-1013p may be a potential therapeutic target for calcific aortic valve disease.

**Supplementary Information:**

The online version contains supplementary material available at 10.1186/s10020-023-00619-4.

## Introduction

Calcific aortic valve disease (CAVD) is a major health problem and the second leading cause of adult heart diseases, especially among the elderly (Freeman and Otto 2005; Baumgartner 2005; Lindroos et al. 1993). More than just a result of degenerative processes, CAVD is now considered to be a complex and active process characterized by multiple pathologic changes (Liu et al. 2007; Miller et al. 2011; Rajamannan et al. 2011). The aortic valve interstitial cell (AVIC) is a fibroblast-like cell type that is multipotent and dominant within the aortic valve to maintain normal valve structure and function throughout life (Monzack and Masters 2011). Aortic valve calcification is believed to involve the differentiation of VICs into either a myofibroblastic or an osteoblast-like phenotype during valve repair and remodeling (Monzack and Masters 2011). Physiological interventions to maintain the balance of VIC differentiation, especially under pathologic stimulations, may thus be beneficial in the prevention or treatment of CAVD. However, due to a lack of therapeutic targets for effective interventions, several medical treatments investigated for slowing or reversing the pathogenic process in CAVD have proven ineffective in clinical trials (Chan et al. 2010; Rossebo et al. 2008; Cowell et al. 2005). Until recently, there is still no medical therapy to prevent or reverse the disease progression; the surgical replacement or implantation of the aortic valve is still the only available treatment and is not suitable for all patients.

Since the first microRNA (miRNA) lin-4 was discovered in 1993 (Lee et al. 1993), miRNAs have been considered to provide an additional level of gene regulation beyond that of transcription factors. miRNAs are a class of small, non-coding RNA molecules that regulate gene expression post-transcriptionally in eukaryotic cells (Bartel 2004; Ambros 2004; Lim et al. 2003). miRNAs are approximately 22 nucleotide, single-stranded RNAs that silence target mRNAs in order to induce target mRNA degradation or translational repression by complementary base-pair binding to the 3′ untranslated regions (3′UTR) (Bartel 2009; Tay et al. 2008). Numerous studies have confirmed that miRNA dysregulation plays important roles in various diseases, such as cancer, cardiovascular diseases, and diabetes, thereby potentiating significant diagnostic and therapeutic approaches (Rossbach 2010; O'Connell et al. 2010; Erson and Petty 2008; Soifer et al. 2007). For example, miRNA mimics and molecules targeted at miRNAs (antimiRs) have shown promise in preclinical development (Rupaimoole and Slack 2017) and over 100 clinical trials treating various diseases (Rupaimoole and Slack 2017; Dai et al. 2015). Several miRNAs have already been found to have important functions in osteo-/chondro-genesis and cartilage/bone formation, such as miR-140 (Le et al. 2013) and miR-101 (Dai et al. 2012, 2015). Recent studies demonstrate that miR-101 plays an active role in the regulation of extracellular matrix (ECM) remodeling including fibrosis (Zhao et al. 2015; Pan et al. 2012), osteoclast differentiation (Lee et al. 2011) and apoptosis (Wu et al. 2015). However, very few studies about the regulatory functions of miRNAs in CAVD have been conducted (Zhang et al. 2014).

miR-101 exacerbated chondrocyte ECM degradation by directly targeting and regulating SOX9 expression, and silencing of miR-101 prevented cartilage degradation in a rat osteoarthritis (OA) model (Dai et al. 2012, 2015). SOX9 is a transcription factor belonging to the Sry-related high-mobility-group box (SOX) protein family (Lefebvre et al. 2007). It is essential for chondrogenesis of MSCs (Guerit et al. 2013; Cairns, et al. 2012; Akiyama 2008), since it is the key transcription factor for BMP2 induced chondrogenesis (Pan et al. 2008). SOX9 is also reported to inhibit the transactivation of Runt-related transcription factor 2 (Runx2) (Cheng and Genever 2010; Yamashita et al. 2009; Zhou et al. 2006), a key transcription factor for osteogenesis, endochondral ossification and aortic valve calcification (Miller et al. 2011; Bruderer et al. 2014; Chen et al. 2014; Towler 2013). Therefore, SOX9 is considered to promote chondrogenesis and prevent osteogenesis. Since both osteogenic and chondrogenic ossification (or bone formation) are present in CAVD (Rajamannan et al. 2011; Mohler et al. 2001), reduced SOX9 promotes aortic valve calcification (Peacock et al. 2010; Lincoln et al. 2007), whereas SOX9 overexpression induces chondrogenesis, which is regulated by the Wnt/β-catenin signaling (Fang et al. 2014).

Cell–cell adhesion generated by cadherins plays an important role in multiple aspects of cellular behavior including proliferation, differentiation, apoptosis and the maintenance of tissue integrity (Cavallaro and Dejana 2011; Harris and Tepass 2010). Cadherin-11 (CDH11) is a cell–cell adhesion protein that regulates the differentiation of mesenchymal cells into the osteo- and chondro-lineages (Kii et al. 2004). CDH11 plays an important role in the aortic valve maturation through coordinating cellular migration and extracellular matrix remodeling (Bowen et al. 2015; Zhou et al. 2013); whereas the dysregulation of CDH11 impairs the aortic valve (AV) structural integrity and promotes calcific nodule development on the AVs or in AVICs (Bowen et al. 2015; Sung et al. 2016; Hutcheson et al. 2013).

In this study, we identified a mechanism by which miR-101-3p contributes to human aortic valve interstitial cells (HAVICs) calcification via direct downregulation of SOX9 and CDH11 and activation of osteogenesis. We demonstrated that the expression of miR-101-3p is significantly increased in calcified human aortic valves, suggesting that it is a potential regulator of osteogenesis/chondrogenesis in HAVICs. Using mimic and anti-miR of miR-101-3p, we found that the miR-101-3p mimic promotes HAVICs calcification, but the inhibition of miR-101-3p efficiently protects HAVICs from calcification even in the osteogenic-conditioned medium. We determined that miR-101-3p directly targets CDH11 and SOX9 expression, and that inhibition of miR-101 prevents osteogenic differentiation in HAVICs by restoring SOX9 and CDH11 expressions. These results suggest that inhibition of miR-101 is a potential therapeutic strategy for CAVD.

## Materials and methods

Expended methods can be found in Additional file 1.

### RNA isolation from human aortic valves, small RNA deep sequencing and qPCR analysis

RNA isolation, small RNA sequencing and target prediction and qPCR were performed as we described recently (Chen et al. 2021a, 2022; Chen and Sun 2019). See Additional file 1: Methods.

### Isolation and culture of primary human aortic valve interstitial cells

Human aortic valve interstitial cells (HAVICs) were isolated as described in our previous study (Chen et al. 2016; Song et al. 2015).

### miRNA/siRNA transfection and in vitro calcification of HAVICs

The miRNA/siRNA transfection procedure was performed as we described previously (Chen et al. 2021a; Wang et al. 2021) See Additional file 1: Methods.

### Western blot analysis

The western blot procedure was performed as described in our recent study (Wang et al. 2021; Chen et al. 2021b; Chen and Sun 2021; Han and Sun 2020, 2022).

### Human aortic valve histology and immunohistochemistry staining

The histological and immunohistochemical (IHC) procedures were performed as described in our previous studies (Chen et al. 2016; Lin et al. 2016; Fan et al. 2022a, b).

### Statistical analysis

Quantitative data were presented as the Means ± SEM. The unpaired t-test was used for comparisons between two groups. Differences between experimental groups were examined by one-way analysis of variance (ANOVA), followed by the Tukey post-test or two-way ANOVA, followed by the Bonferroni post-test using Prism software (GraphPad). For all analyses, p < 0.05 was considered statistically significant.

## Results

The data that support the findings of this study are available upon reasonable request from the corresponding author.

### miR-101 expression increased in the calcified human aortic valves

**Fig. 1:**
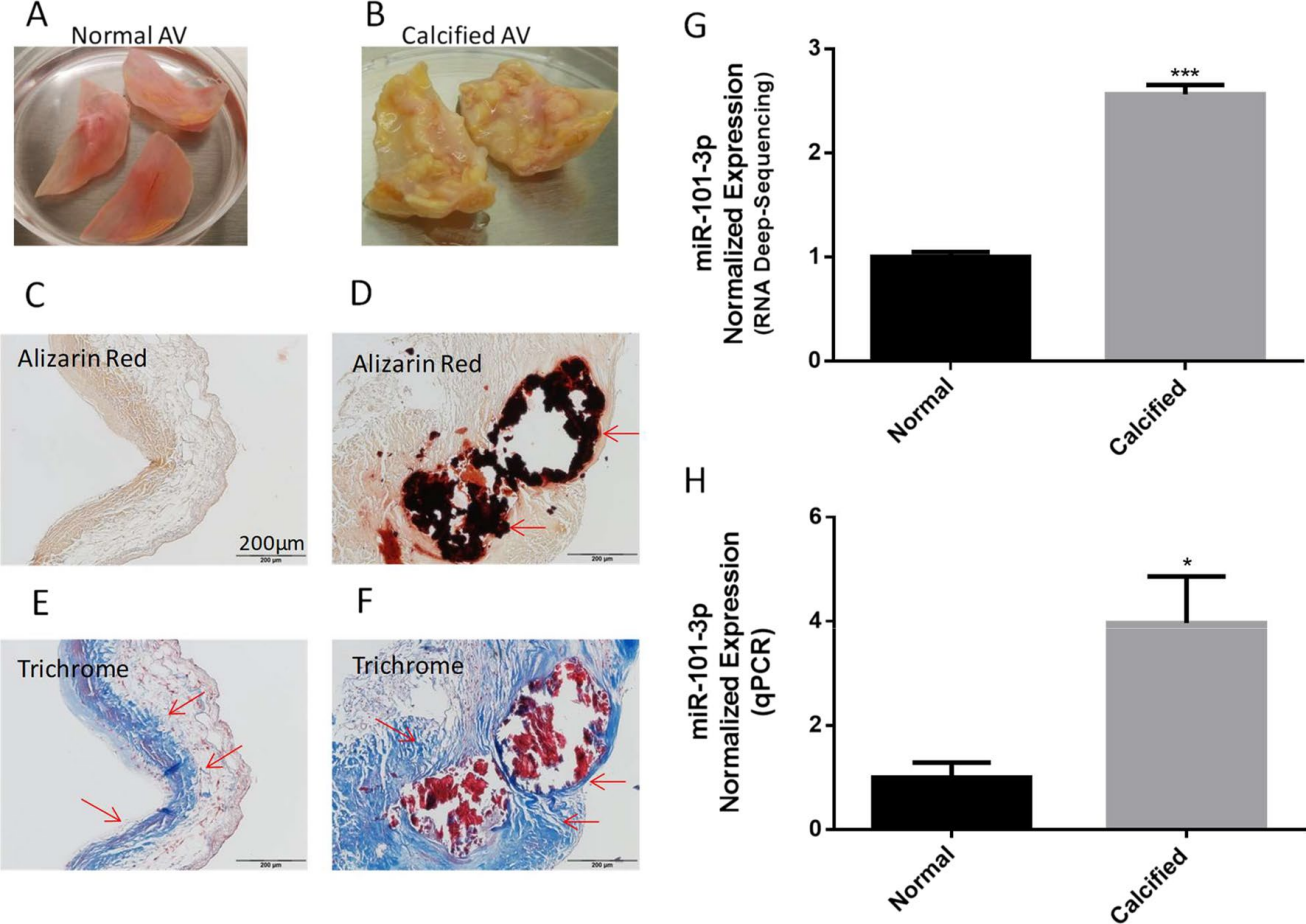
miR-101-3p expression was upregulated in the calcified human aortic valves. **A**, **B** Photomicrographs of freshly isolated normal and calcified human aortic valves. **C**, **D** Alizarin red staining of normal and calcified human aortic valves, red staining indicating calcification (indicated by arrows). **E**, **F** Masson’s Trichrome staining of collagen deposition (blue staining indicated by arrows) of normal and calcified human aortic valves. **G** Small RNA deep sequencing results of miR-101-3p of normal and calcified human aortic valves. Data = means ± SEM. ***P < 0.001 vs Normal, n = 3. **H** qPCR analysis of miR-101-3p of normal and calcified human aortic valves. Scale bar = 200 μm. *P < 0.05 vs Normal, n = 3

Figure 1A, B illustrate the dramatic structural and morphologic changes in calcified human aortic leaflets vs. the age-matched normal human aortic valve leaflets. Calcified leaflets developed an osseous structure. Alizarin red staining (Fig. 1C, D) displayed a significant calcium deposition on the calcified valves. Masson’s trichrome staining of collagen (blue color) indicated that calcium deposition occurred in the collagen-rich region close to the aortic side of the calcified valves (Fig. 1E, F).

Using the small RNA-Seq technology we generated microRNA-profiling libraries in normal and calcified aortic valve cells. There are hundreds of mRNA expressions up or down regulated (threshold > 1.5) in the calcified human aortic valves. The small RNA-Seq analysis showed that among these mRNA expressions, miR-101-3p levels in the calcified human aortic valves were almost 3 times higher than that of normal HAVs (Additional file 1: Fig. S1, Fig. 1G). Based on target prediction analysis of top miRs, we found that miR-101-3p may target Cadherin-11 (CDH11) and SOX9 (Additional file 1: Fig. S2) which are involved in chondrogenesis and osteogenesis. The qPCR analysis further confirmed that in the calcified HAVs, the miR-101-3p expression level was increased significantly (Fig. 1H). Therefore, we investigated whether miR-101-3p may contribute to human aortic valvular interstitial cell (HAVIC) calcification in isolated HAVICs.

### miR-101-3p played a role in calcific nodule development in the HAVICs

**Fig. 2:**
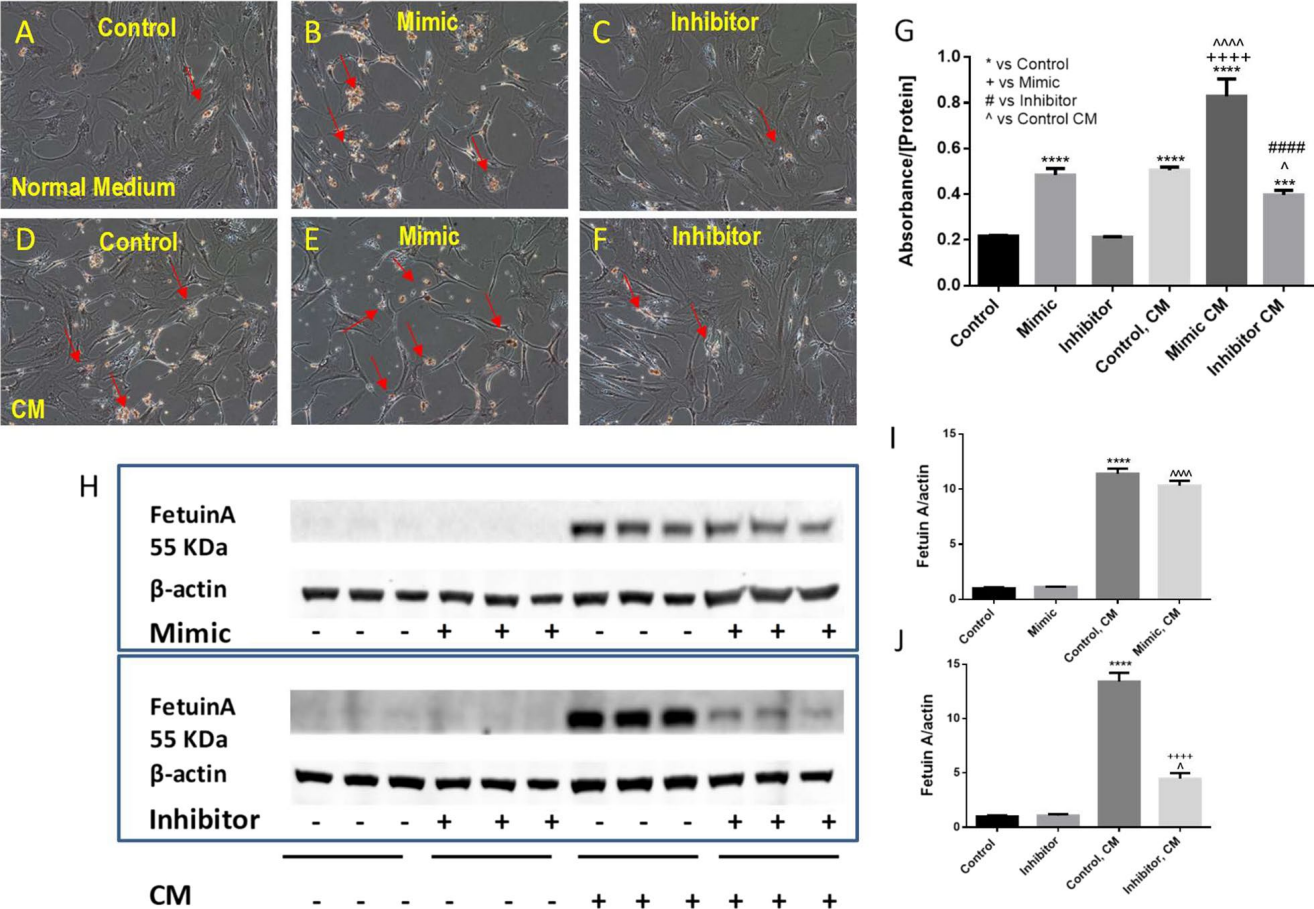
miR-101-3p mimic promoted HAVICs calcification and inhibition of miR-101-3p protected HAVICs from calcification in CM. Alizarin red staining (**A**–**F**) of HAVICs cultured in osteogenic-conditioned media (10 mM βGP, 5 mM CaCl_2_) for 14 days after transfected with miR-101-3p mimic, inhibitor and negative control. **G** Quantification of alizarin red staining, absorbance normalized by protein concentration. *P < 0.05, ***P < 0.001 and ****P < 0.0001 vs Control; ^++++^P < 0.0001 vs Mimic; ^P < 0.05, ^^^^P < 0.0001 vs Control, CM; ^####^P < 0.0001 vs Inhibitor. **H** Western blot analysis of Fetuin A protein expression in HAVICs cultured in CM for 48 h after miR-101 mimic/inhibitor transfection. β-actin serves as internal loading control. **I**, **J** Quantification of Fetuin A protein expression normalized by β-actin. Data = means ± SEM. ****P < 0.0001 vs Control; ^P < 0.05 and ^^^^P < 0.0001 vs Mimic or Inhibitor groups; ^++++^P < 0.0001 vs Control, CM. n = 3 repeats

In order to investigate whether miR-101-3p plays a role in HAVIC calcification, we transfected HAVICs with miR-101-3p mimic and inhibitor sequences in the presence of normal and osteogenic-conditioned medium. The osteogenic-conditioned medium induces calcification in vitro. Alizarin red staining indicated that miR-101 mimic alone promoted calcific nodule development in HAVICs even in normal medium (Fig. 2B). In the conditioned medium, calcific nodules developed in the HAVICs transfected with control miR, (Fig. 2D) while the number of calcified nodules were further increased in the HAVICs transfected with miR-101 mimic (Fig. 2E). This result suggests that miR-101 mimic may promote HAVIC calcification. However, the calcified nodule formations were significantly decreased in the HAVICs transfected with miR-101-3p inhibitors (Fig. 2F), suggesting that the inhibition of miR-101 may suppress HAVIC calcification. The quantification of absorbance of alizarin red staining normalized by protein concentration demonstrated that miR-101-3p mimic promoted calcium deposition in the HAVICs in both normal and conditioned medium, and that miR-101-3p inhibitor prevented HAVICs from calcification in the conditioned medium (Fig. 2G). Western blot analysis of Fetuin A, a calcium-binding protein and an early calcification marker, also showed that Fetuin A was significantly increased in the HAVICs in CM (Fig. 2H, I), and that inhibition of miR-101 decreased Fetuin A level in HAVICs (Fig. 2H, J). Collectively, these results suggest that inhibiting miR-101 protects HAVICs from inducible calcification and that miR-101 promotes calcific nodule development within them.

### miR-101 promoted osteogenesis in HAVICs through regulation of CDH11 and SOX9 expression

**Fig. 3:**
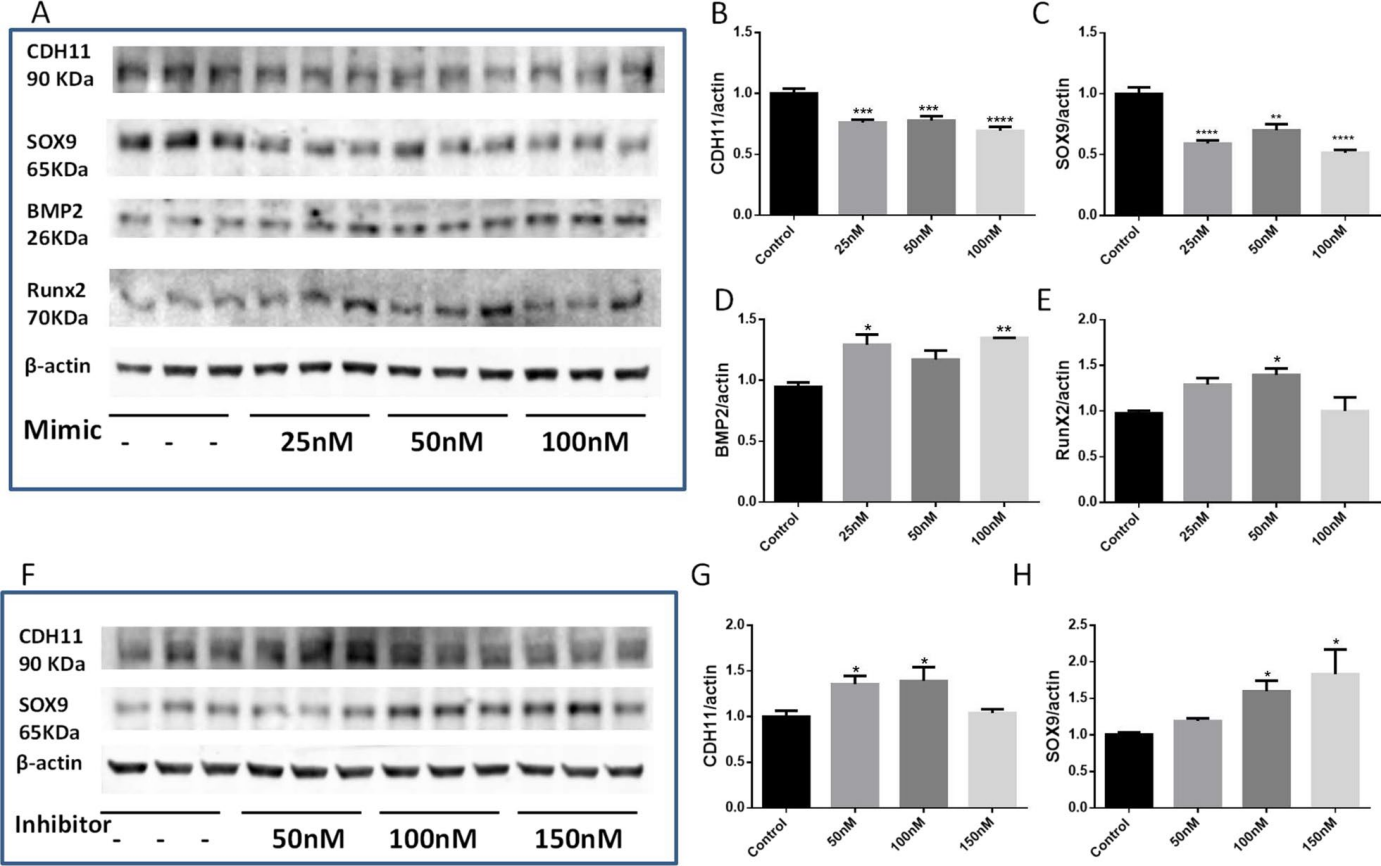
miR-101-3p regulated CDH11 and Sox9 protein expression and osteogenesis markers BMP2/Runx2 in HAVICs. **A** Western Blots against antibodies of Cadherin-11 (CDH11) (ab147215), Sox9 (AB5535, Millipore), BMP2 (ThermoFisher, 710022), and Runx2 (Santa Cruz sc-12488) in HAVICs at 72 h after transfection with negative control and various doses of miR-101-3p mimic. **B**–**E** Quantification of CDH11, Sox9, BMP2, and Runx2 protein expression normalized with β-actin. **F** Western Blots against antibodies of Cadherin-11 (CDH11) and Sox9 in HAVICs at 72 h after transfection with negative control and various doses of mir-101-3p inhibitor. **G**, **H** Quantification of CDH 11 and Sox9 protein expression normalized with β-actin. Data = means ± SEM. *p < 0.05, **P < 0.01, ***P < 0.001 and ****P < 0.0001 vs Control. n = 3 repeats

We further investigated how miR-101 promotes calcification in HAVICs. Western blot analysis showed that expression of osteogenesis markers BMP2 and Runx2 displayed dose-dependent increases after HAVICs are transfected with various doses of miR-101 mimic (Fig. 3A, D, E). Using TargetScan Human (Release 7.1) (Chen et al. 2021a; Agarwal et al. 2015), we found that mRNAs of BMP2 and Runx2 do not have any predicted binding site of miR-101. Thus, we postulate that there may be upstream factors that are potentially direct targets of miR-101 and that mediate the role of miR-101 in regulating BMP2 and Runx2 expression in HAVICs.

Among the hundreds of potential mRNA targets of miR-101 (TargetScan Human, Release 7.1) (Chen et al. 2021a; Agarwal et al. 2015), Cadherin-11 (CDH11) and SOX9, which are potentially involved in ECM remodeling, caught our attention. Both of them had two 7-mer miR-101-3p binding sites on their mRNAs (Additional file 1: Fig. S2). Western blot analysis showed the decreased protein expressions of CDH11 and SOX9 in HAVICs after miR-101-3p mimic transfection (Fig. 3A-C), whereas inhibition of miR-101-3p by transfection of miR-101 inhibitor (anti-miRNA-101) increased CDH11 and SOX9 protein levels in HAVICs (Fig. 3F–H). These results provide the first evidence that miR-101 directly regulates CDH11 and SOX9 gene expression in HAVICs. Collectively, all these data suggest that upregulation of miR-101 in the calcified human aortic valves may play an important role in promoting CAVD development via targeting the protein expression of CDH11 and SOX9.

**Fig. 4:**
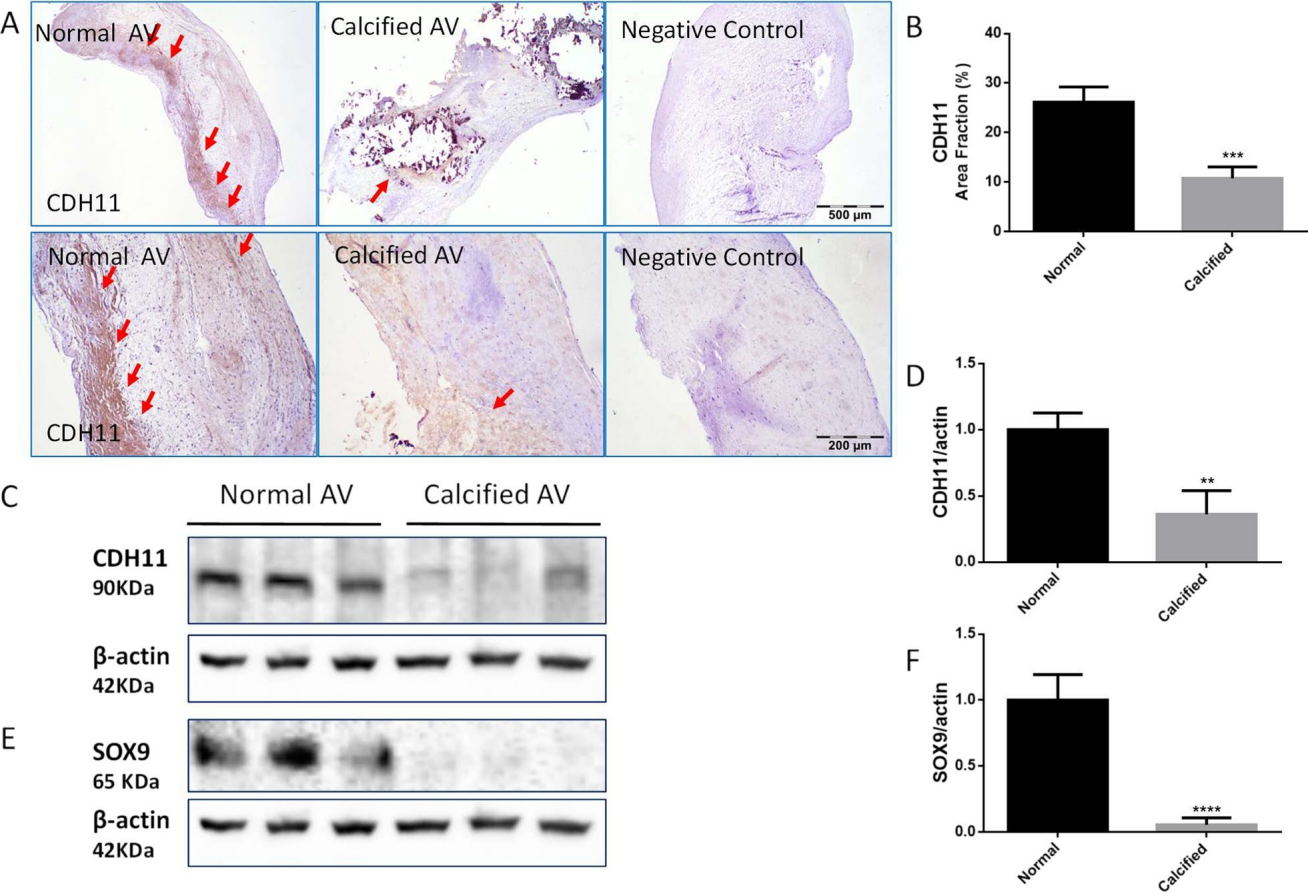
CDH11 and SOX9 protein expression were decreased in calcified human aortic valves (HAVs). **A** Immunohistochemical staining against the antibody of Cadherin-11 (CDH11) (ab147215) on calcified human aortic valves and age-matched normal human aortic valves (Positive staining indicated by red arrows). Negative control staining without the secondary antibody. **B** Semi-quantification of area fraction of CDH11 staining on human aortic valves. ***P < 0.001 vs Normal. **C**, **D** Western blot analysis of CDH11 protein expression in human aortic valves normalized with β-actin. **E**, **F** Western blot analysis of SOX9 protein expression in human aortic valves normalized with β-actin. In **A**, scale bar = 500 μm for the upper panel and 200 μm for the lower panel. Data = means ± SEM. **P < 0.01, and ****P < 0.0001 vs Normal. n = 3

We next assessed CDH11 and Sox9 expression in the calcified human aortic valves (HAVs). Immunohistochemical analysis indicated that CDH11 expression was decreased in calcified HAVs (Fig. 4A, B). Western blot analysis further confirmed downregulation of CDH11 protein expression in calcified human aortic valves (Fig. 4C, D), as well as SOX9 protein expression (Fig. 4E, F).

### CDH11 and Sox9 regulated osteogenesis

Knockdown of CDH11 upregulated SOX9 expression in HAVICs (Additional file 1: Fig. S3A–C). Interestingly, knockdown of SOX9 increased CDH11 expression (Additional file 1: Fig. S3D–F). These results suggest that CDH11 and SOX9 may cross-regulate each other. Knockdown of CDH11 or SOX9 alone was insufficient to induce osteogenesis (Additional file 1: Fig. S4A–C). In contrast, knockdown of both CDH11 and SOX9 together promoted osteogenesis as evidenced by upregulation of Runx2, BMP2 and OCN expression in HAVICs (Additional file 1: Fig. S5).

### miR-101-3p regulated osteogenesis in HAVICs treated with osteogenic-conditioned medium (CM)

**Fig. 5:**
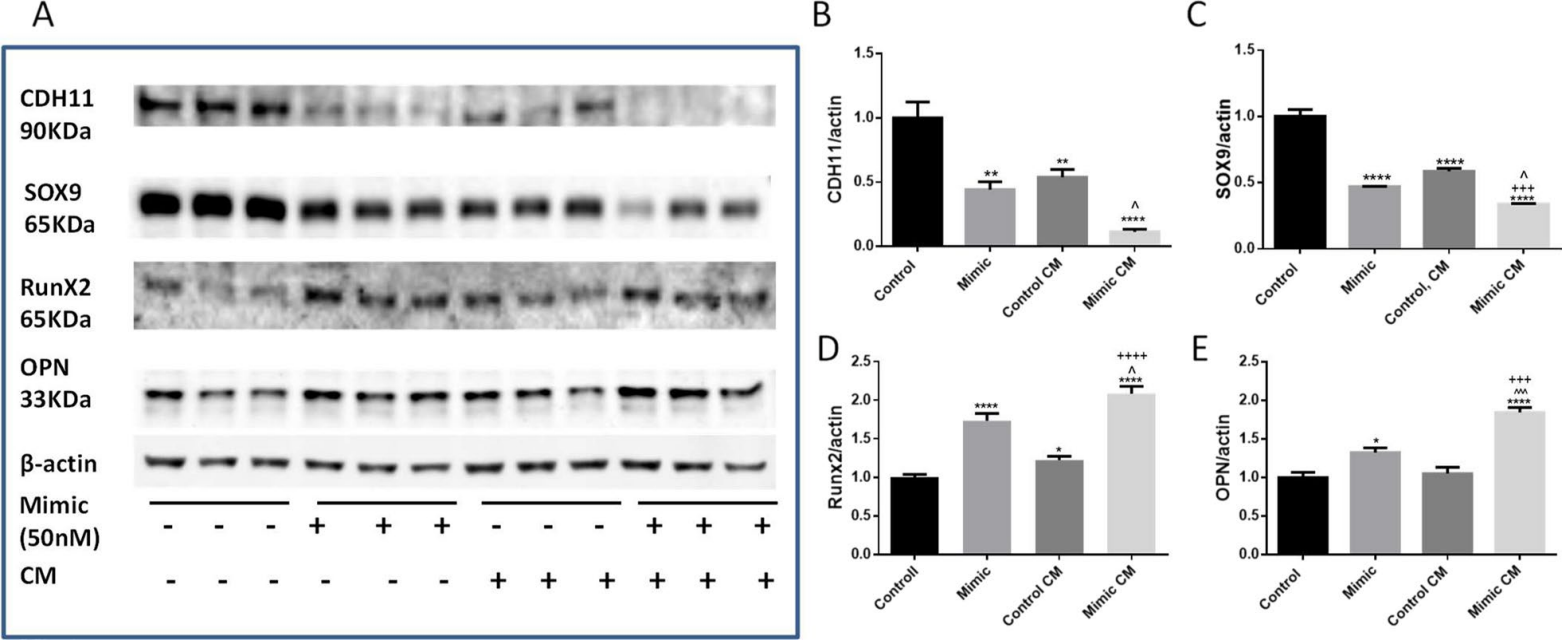
Mimic of miR-101 downregulated CDH-11 and SOX9 expression and promoted osteogenesis in HAVICs treated by CM after transfection. **A** Western blot against antibodies of CDH11, SOX9, Runx2, and OPN in HAVICs transfected with miR-101-3p mimic for 48 h and then treated with CM for additional 48 h. β-actin serves as internal loading control. **B**–**E** Quantification of CDH11, SOX9, Runx2, and OPN normalized with β-actin. Data = means ± SEM. *p < 0.05, **P < 0.01 and ****P < 0.0001 vs Control; ^+++^P < 0.001 vs Control, CM; ^P < 0.05 and ^^^P < 0.001 vs Mimic. n = 3 repeats

In order to induce in vitro calcification in HAVICs, cells are usually treated with conditioned medium (10 mM βGP and 5 mM CaCl_2_ in M199) (Yang et al. 2009). To investigate the functional role of miR-101-3p in the regulation of calcification in HAVICs, we transfected HAVICs with miR-101 mimic and inhibitor for 48 h and then treated cells with CM for another 48 h. Western blot analysis showed significant decreases in CDH11 and SOX9 protein expression in miR-101 mimic-treated cells cultured in CM; HAVICs transfected with mimic and cultured in CM had lowest protein expression levels of CDH11 and SOX9 in all groups (Fig. 5A–C). Most importantly, transfection with miR-101 mimic upregulated osteogenesis markers Runx2 and OPN in HAVICs. Culturing with CM further increased protein expression of Runx2 and OPN in HAVICs (Fig. 5A, D, E). Overall, these data suggest that miR-101 promoted osteogenesis and calcification likely through direct targeting CDH11 and SOX9 mRNAs.

**Fig. 6:**
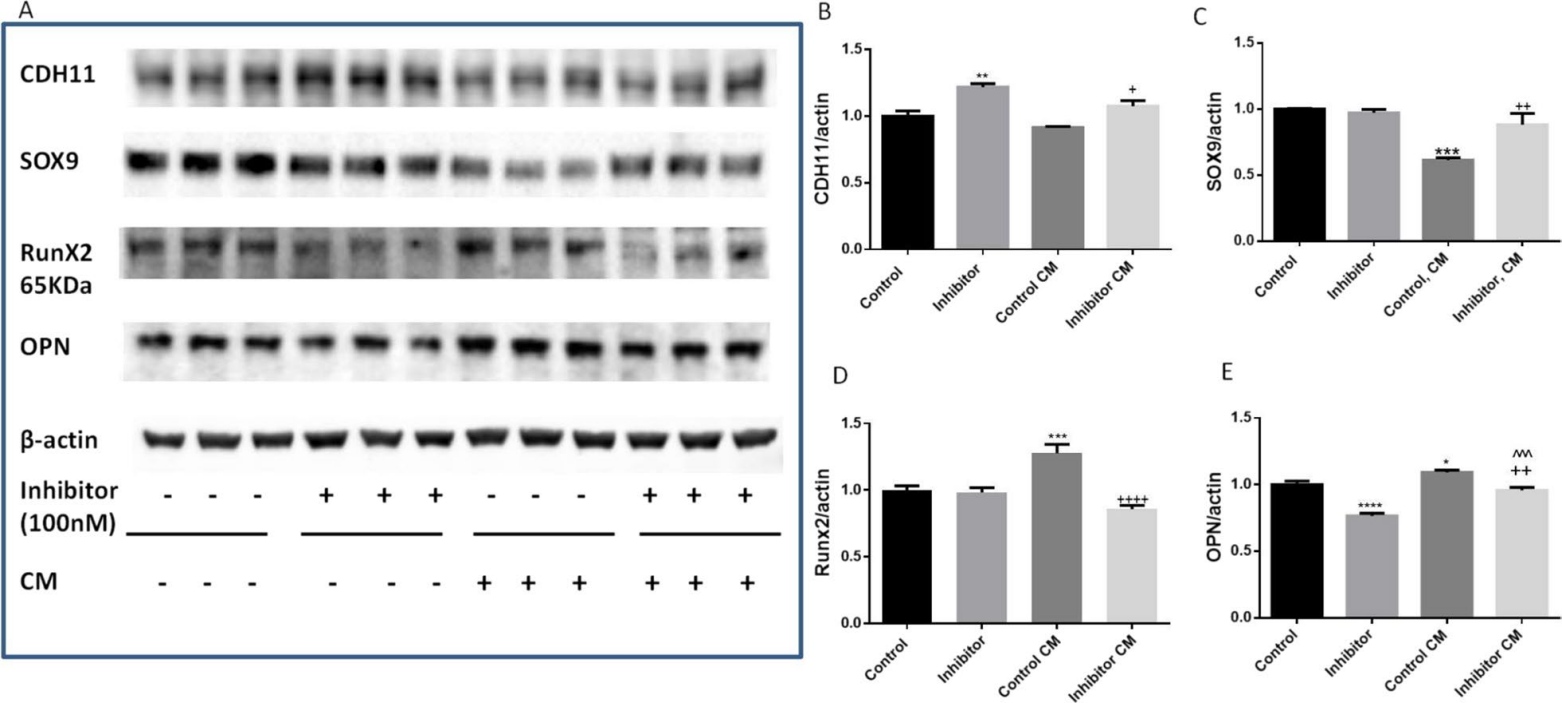
Inhibition of miR-101 rescued downregulation of CDH-11 and SOX9 expression and prevented osteogenesis in CM-treated HAVICs. **A** Western blot against antibodies of CDH11, SOX9, Runx2, and OPN in HAVICs transfected with miR-101-3p inhibitor for 48 h and then treated with CM for additional 48 h. β-actin serves as internal loading control. **B**–**E** Quantification of CDH11, Sox9, Runx2 and OPN protein expression normalized with β-actin. Data = means ± SEM. **P < 0.01 and ***P < 0.001 vs Control; ^+^P < 0.05, ^++^P < 0.001 and ^++++^P < 0.0001 vs Control, CM. n = 3 repeats

Conversely, transfection with miR-101 inhibitor (anti-miR-101) rescued the downregulation of CDH11 and SOX9 expression levels in the CM, suggesting the inhibition of miR-101-3p in CM protected CDH11 and SOX9 expression from in vitro osteogenic induction (Fig. 6A–C). Transfection with miR-101 inhibitor also abolished upregulation of Runx2 and OPN protein expression (Fig. 6A, D, E) in CM. Overall, these results suggest that miR-101-3p is actively involved in the regulation of osteogenesis in HAVICs and that inhibition of miR-101-3p is efficient in protecting HAVICs from osteogenic differentiation and calcification in vitro.

By theory, the miR-101-3p inhibitor should inhibit miR-101-3p which increases SOX9 protein expression. However, the miR-101-3p inhibitor did not affect the basal SOX9 protein expression level. This is likely because the basal SOX9 protein expression is high (Fig. 6C), inhibition of miR-101-3p did not further increase its expression. As a result, the basal protein expression of RUNX2, a target of SOX9, was not affected by miR-101-3p inhibitor (Fig. 6D). Nevertheless, the miR-101-3p inhibitor prevented CM-induced downregulation of SOX9 protein expression and, consequently, upregulation of RUNX2 protein expression. In contrast, Fig. 5C, D showed that miR-101-3p mimic downregulated the basal level of SOX9 protein expression which resulted in an increase in Runx2 expression.

### miR-101 regulated asporin (ASPN) expression in HAVICs treated with osteogenic-conditioned medium

**Fig. 7:**
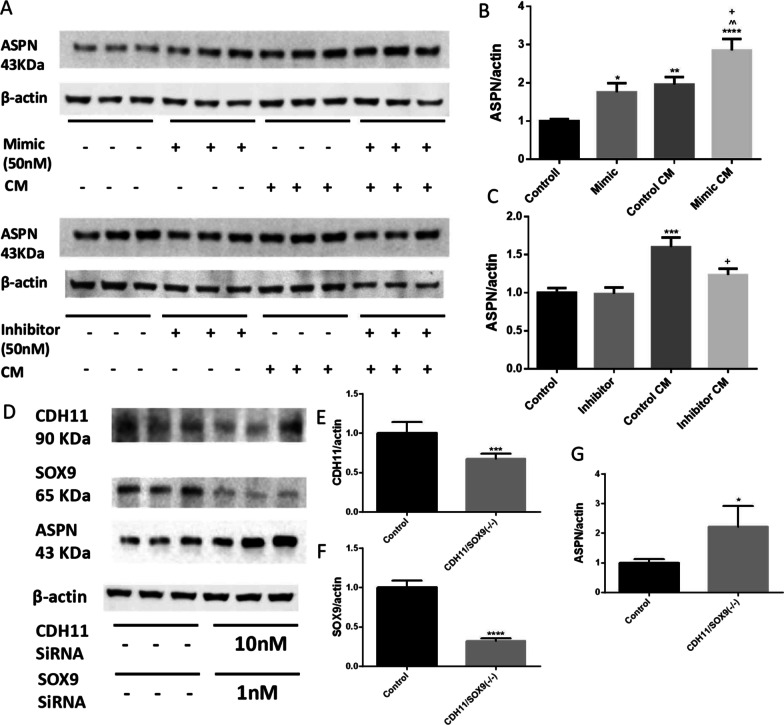
miR-101 mediated ASPN expression in CM treated HAVICs through regulation of CDH11 and Sox9. **A** Western Blot against antibodies of ASPN in HAVICs transfected with miR-101-3p mimic or inhibitor for 48 h and then treated with CM for 48 h. β-Actin serves as internal loading control. **B**, **C** Quantifications of ASPN protein expression normalized with β-actin. *P < 0.05 and ****P < 0.0001 vs Control; ^+^P < 0.05 and ^++^P < 0.01 vs Control, CM; ^^P < 0.01 vs Mimic. **D** Western Blot against CDH11, Sox9 and ASPN in HAVICs co-transfected with SiRNAs of CDH11 (10 nM) and Sox9 (1 nM) for 72 h. **E**–**G** Quantifications of WBs normalized by β-actin. Data = means ± SEM. ***P < 0.001 and ****P < 0.0001 vs Control. n = 3 repeats

Asporin was reported as a regulator of osteoblast mineralization (Kalamajski et al. 2009; Tomoeda et al. 2008) and played regulatory roles in the chondrogenesis (Nakajima et al. 2007). Interestingly, a significant increase of ASPN expression was found in the HAVICs cultured in the CM as well as in HAVICs treated with miR-101-3p mimic (Fig. 7A, B). ASPN expression levels in the mimic-treated HAVICs cultured in CM were almost threefold higher than those in the control HAVICs. On the other hand, inhibition of miR-101 in the CM-treated HAVICs decreased the ASPN expression level to the normal level (Fig. 7A–C). These results suggest that miR-101 regulates ASPN in HAVIC calcification. Interestingly, siRNAs knockdown of CDH11 or SOX9 separately in the HAVICs did not affect ASPN expression (Additional file 1: Fig. S4C). However, knockdown of both proteins via co-transfection of CDH11 and SOX9 SiRNA led to a significant increase of ASPN protein expression in HAVICs (Fig. 7D–G, Additional file 1: Fig. S5A, B). This result suggests that miR-101 upregulates ASPN expression in HAVICs likely through CDH11 and SOX9. These results provided new mechanistic insights into the regulation of ASPN, a regulator of osteogenesis and calcification (Kalamajski et al. 2009; Tomoeda et al. 2008).

## Discussion

CAVD is considered a progressive process, and the aortic valve interstitial cells (AVICs) are closely regulated by multiple levels of biological factors in various physiologic scenarios, facing numerous stresses and challenges during an entire lifespan to keep the structural and functional integrity of the aortic valves. However, the mechanisms of the development of CAVD remain elusive due to the complicated cell potency of AVICs, which have the ability to differentiate into other type-like cells, such as osteoblast-like and osteoclast-like cells under various circumstances.

The present study demonstrates that increased expressions of miR-101-3p plays a crucial role in the regulation of HAVIC calcification. To the best of our knowledge, this is the first report showing that miR-101 directly promotes HAVIC calcification. More importantly, the inhibition of miR-101-3p effectively prevented or attenuated the calcification in HAVICs even under osteogenic conditions (Fig. 2). This finding not only provides new therapeutic insights into CAVD, but also fundamentally advances the current understanding of the molecular regulation of CAVD. Furthermore, the current study reveals novel mechanisms through which miR-101-3p regulates HAVIC calcification. Specifically, miR-101-3p directly binds to CDH11 and SOX9, which promotes osteogenesis. All these data suggest that inhibition of miR-101-3p in the HAVICs may offer a useful strategy for effective protection of aortic valves from calcification under osteogenic stress.

Aortic valve fibrosis and calcification share distinct processes in ECM remodeling which involves regulations through different signaling pathways. However, there are crosstalk and autoregulation of these pathways, which are mediated by unknown regulators (proteins). It has been reported that CDH11 and SOX9 are associated with ECM remodeling in the aortic valves; therefore, dysregulation of these proteins may promote osteogenesis and calcification of AVs (Peacock et al. 2010; Lincoln et al. 2007; Kii et al. 2004; Bowen et al. 2015; Sung et al. 2016). We have observed that in HAVICs, miR-101-3p activated the osteogenic pathway and promoted calcification (Fig. 5). Our data also demonstrate that the miR-101-3p mimic downregulated expression of CDH11 and SOX9 probably through targeting their mRNAs. Furthermore, our data suggests that siRNA knockdown of CDH11 increased SOX9 expression, and vice versa (Additional file 1: Fig. S3). Thus, CHD11 and SOX9 may cross-regulate each other in mediating HAVIC calcification. We also found that knockdown of either of these two genes alone using SiRNAs was insufficient to promote osteogenesis in the HAVICs (Additional file 1: Fig. S4). Interestingly, knockdown of both CDH11 and SOX9 simultaneously induced osteogenic responses (e.g., upregulation of BMP2, Runx2, ASPN and OCN) (Additional file 1: Fig. S5). Although the mechanism underlying the regulation of SOX9 expression by CHD11 is unclear, it appears that downregulation of both is required for promoting osteogenesis in HAVICs. A limitation of this study is that it lacks mechanistic investigation into the crosstalk of CDH11 and SOX9. A further study is warranted for investigating whether the cell adhesion molecule CDH11 inhibits SOX9 transcription or promotes degradation of SOX9 protein in HAVICs. Collectively, all these findings suggest that the miR-101-3p promotes osteogenesis and calcification in the HAVICs likely via downregulation of CDH11 and SOX9, the major regulators of ECM remodeling, calcification and ossification in aortic valves.

Asporin (ASPN) plays an important role in the regulation of cartilage development and osteoarthritis (OA) pathology (Kalamajski et al. 2009; Tomoeda et al. 2008). ASPN was present in the HAVICs and the expression level was upregulated in the HAVICs treated with osteogenic-conditioned medium (Fig. 7). Knockdown of both CDH11 and SOX9 increased ASPN expression. Thus, mR-101 regulates ASPN expression, likely via CDH11/SOX9.

It was reported that miR-101-3p regulates the PTEN/AKT axis in trophoblast cells in the placenta (Zhang et al. 2022). Metformin can inhibit AVICs calcification in vitro by activating the PI3K/AKT signaling pathway (En et al. 2021). Yang et al. (2022) found that LEVs might inhibit neuron apoptosis via the miR-101a-3p/c-Fos/TGF-β axis, and has-miR-101-3p is a potential marker of neurological recovery in IS patients. TGF-beta1 is characteristically present within calcific aortic stenosis cusps, and mediates the calcification of aortic valve interstitial cells in culture through mechanisms involving apoptosis (Jian et al. 2003). A further study is needed to investigate whether these pathways regulate CDH11, SOX9 and ASPN in AVICs and mediate the role of miR-101-3P in AVIC calcification.

## Conclusion

The present study demonstrates that the level of miR-101-3p was remarkably increased in the calcified human aortic valves, and the miR-101-3p mimic promotes osteogenesis and calcification in the HAVICs (Additional file 1: Fig. S6). Inhibition of miR-101-3p showed strong therapeutic potential to prevent or attenuate the development of HAVIC calcification. Furthermore, the data indicated that miR-101-3p directly targeted CDH11 and SOX9 to regulate ASPN and the downstream osteogenesis pathway. These findings not only advance our current understanding of the mechanism of human CAVD but also provide new therapeutic insights into CAVD. Whether ASPN is directly and actively involved in HAVIC calcification induced by CM and/or miR-101 treatment warrants further investigation.

## Supplementary Information

**Additional file 1.** Online Supplemental Methods and Data.

## Data Availability

The data that support the findings of this study are available upon reasonable request from the corresponding author.

## Abbreviations

CAVD: Calcific aortic valve disease
HAVICs: Human aortic valve interstitial cells
CDH11: Cadherin-11
CHAVs: Calcified human aortic valves
ASPN: Asporin
Runx2: Runt-related transcription factor 2
BMP2: Bone morphogenetic protein 2
OPN: Osteopontin
OCN: Osteocalcin
