# Supplementary material for: Inhibition of miR-101-3p prevents human aortic valve interstitial cell calcification through regulation of CDH11/SOX9 expression
Source: Mol Med. 2023 Feb 21;29:24. doi: 10.1186/s10020-023-00619-4 (PMC9945614; doi:10.1186/s10020-023-00619-4)
Supplement: Supplementary file 1 — Additional file 1. Online Supplemental Methods and Data. [file 10020_2023_619_MOESM1_ESM.pdf]

Additional file 1

Inhibition of miR-101-3p prevents human aortic valve interstitial cell calcification through regulation of CDH11/SOX9 expression

Jianglei Chen[1], Yi Lin[1], Zhongjie Sun[1,2*]

[1]Department of Physiology, College of Medicine, University of Oklahoma Health Sciences Center, Oklahoma City, OK73104, USA

[2]Department of Physiology, College of Medicine, University of Tennessee Health Science Center, Memphis, TN 38163, USA

Running Title: miR-101-3p and CAVD

*Address Correspondence to:

Zhongjie Sun, MD, PhD, FAHA
Professor and Chair
Department of Physiology
College of Medicine
Director, UT Cardiovascular Institute
University of Tennessee Health Science Center
956 Court Avenue
Memphis, TN 38163, USA
Zsun10@uthsc.edu
Tel. 901-448-2679

Additional Methods

RNA isolation from human aortic valves, small RNA deep sequencing and qPCR analysis

This project was approved by the Institutional Review Board (IRB) of the University of Oklahoma Health Sciences Center. Calcified human aortic valves (CHAVs) were obtained from patients who had undergone aortic valve transplantations; normal HAVs were collected from donors who died from non-valve related illnesses with no visible valve pathology. All these valves were provided by NDRI (National Disease Research Interchange, Philadelphia, PA). The patients' information was de-identified.

Total RNA was extracted with Direct-zol^{TM} RNA MiniPrep (ZYMO Research) according to the manufacturer's standard protocol. miRNA libraries were constructed using New England Biolabs (NEB) NEBNext Multiplex Small RNA Library Prep Set for Illumina sequencers and the NEB standard protocol. Individual libraries were constructed using 1μg of total RNA isolated from each sample. Each library was indexed in order to multiplex four to six samples per sequencing run on the Illumina MiSeq platform using MiSeq 50 cycle Reagent Kits v2. A minimum of 15 million 50bp sequencing reads were collected from each sample and data were analyzed using Genesifter software from Perkin Elmer (formerly Geospiza).

Raw data for each sample were aligned to the most recent mirBASE database with remaining reads aligned to the most recent mouse genome reference build in order to identify previously unknown regions that may encode for unique miRNAs. Pairwise comparison of the alignment results was done using Genesifter for identification of miRNAs that are differentially expressed at a significant level, i.e. upregulated or downregulated, in similarly treated samples. Identified miRNAs will be investigated further for their role in disease.

Quantitative PCR (qPCR) of miR-101 was performed *via* real-time PCR, using TaqMan MicroRNA Assays (Thermal Fisher) by following the manufacturer's protocol. Expression data were normalized to RNU48 (TaqMan microRNA control, Thermal Fisher) as internal control for human aortic valves.

Isolation and culture of primary human aortic valve interstitial cells

HAVICs were cultured in M199 growth medium supplemented with 10% fetal bovine serum (FBS), 1% penicillin/streptomycin, and amphotericin B. Cultures were refreshed every 48 h and split 1:3 at confluence. Cultures were used for experiments between passages 6-8.

miRNA/siRNA transfection and *in vitro* calcification of HAVICs

HAVICs were incubated to 80% confluence in a 6-well plate and then transfected with miR-101 mimic, inhibitor and mirVana negative control (10 nM), respectively, purchased from mirVana miRNA, Thermo Fisher or Trilencer-27 Human siRNA of CDH11 and SOX9 (Origene, Rockville, MD) using Lipofectamine RNAiMAX (Thermo Fisher) the following manufacturer's instruction. *In vitro* calcification was induced by the presence of β-glycerolphosphate (10mM) and $CaCl_2$ (5mM) for at least 48 hours. Calcification was examined by using Alizarin Red S Indicator (RICCA Chemical Company, TX) and Nikon microscope (Tokyo, Japan). To quantify Alizarin Red staining, the stained cells were incubated in 10% acetic acid for 30 min and absorbance was measured at 405 nm with a microplate reader. All experiments were repeated at least three times for statistical analysis.

MirVana miRNA inhibitors are small, chemically modified, single-stranded RNA molecules designed to specifically bind to and inhibit endogenously miRNA function by

downregulation of miRNA activity. The detailed information about mirVana miR-101 inhibitor (assay ID MH11414) can be found in mirVana, ThermoFisher.

Western Blot Analysis

Briefly, HAVs and HAVICs were lysed in RIPA buffer containing a protease inhibitor cocktail and a phosphatase inhibitor cocktail (Research Products International Corp., Mount Prospect, IL USA). The total protein levels were measured using a BCA protein assay kit (Thermo Fisher Scientific). An equal amount of protein was loaded on a 4%–20% ExpressPlus™ PAGE gel (GenScript, NJ), and the proteins were transferred onto nitrocellulose filters after separation in the gel. Blots were blocked in 1% BSA in TBST for 1 hour at room temperature, and the membranes were incubated with a primary antibody at 4°C overnight. The primary antibodies used were FetuinA (Santa Cruz, sc-9668), CDH-11 (abcam, ab174215), SOX9 (Millipore, AB5535), BMP2 (Thermo Fisher , 710022), RunX2 (Santa Cruz, sc-12488), OPN (abcam, ab91655), ASPN (abcam, ab58741), and β-actin (abcam). The membranes were incubated with HRP-conjugated or Alexa 680 secondary antibodies for 1 hour at room temperature. Proteins were visualized, examined, and quantified by densitometry using ChemDoc XRS with Quantity One Software (BioRad, Hercules, and CA). Blots were repeated a minimum of three times for every condition.

Human aortic valve histology and immunohistochemistry staining

Briefly, a series of 5-µm cross-sections of the human aortic valves were cut and stained using the Masson Trichrome Staining Kit (EMS, Hatfield, PA). Calcium deposition was analyzed by using alizarin red staining kit (IHC WORLD, Woodstock, MD) as per the manufacturer's protocol. IHC staining against CDH11 and Sox9 was performed using the ABC staining system (Santa

Cruz Biotechnology). Images of aortic valves were collected at the same exposure conditions under an Olympus DP26 microscope. The fractional areas of positive staining against antibodies (brown, IHC) on the aortic valves were acquired and quantified using image J software (NIH).

Additional Data

A.

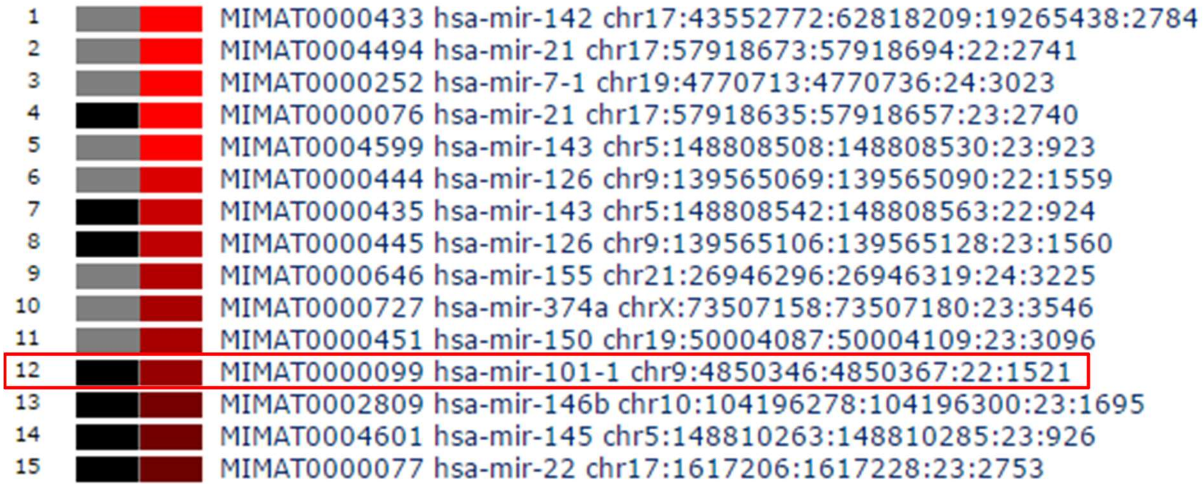

B.

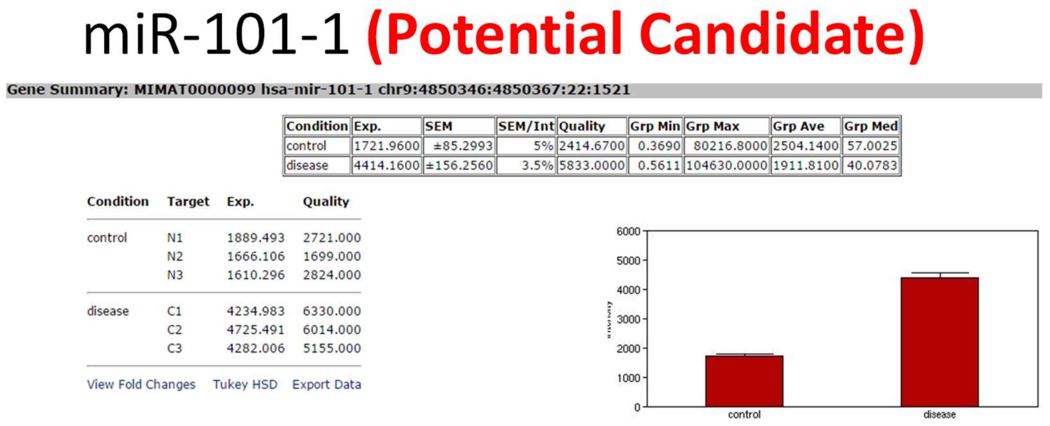

Condition	Exp.	SEM	SEM/Int	Quality	Grp Min	Grp Max	Grp Ave	Grp Med
control	1721.9600	±85.2993	5%	2414.6700	0.3690	80216.8000	2504.1400	57.0025
disease	4414.1600	±156.2560	3.5%	5833.0000	0.5611	104630.0000	1911.8100	40.0783

Condition	Target	Exp.	Quality
control	N1	1889.493	2721.000
	N2	1666.106	1699.000
	N3	1610.296	2824.000
disease	C1	4234.983	6330.000
	C2	4725.491	6014.000
	C3	4282.006	5155.000

>hsa-mir-101-1 MI0000103

UGCCCUGGCUCAGUUAUCACAGUGCUGAUGCUGUCUAUUCUAAAGGUACAGUACUGUGAUAACUGAAGGAUGGCA

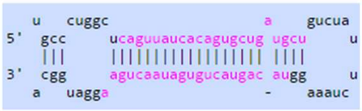

Figure S1. MiRNAs upregulated in HAVICs isolated from calcified human aortic valves. A, Small RNA-Seq analysis of miRNA profiling, threshold>2. **B,** MiR-101 was upregulated in calcified (diseased) valvular cells.

miR101-3p binding sites on Cadherin 11 : 7mer-m8

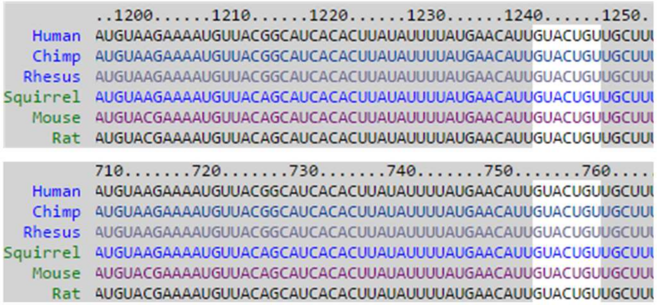

Sequence of miR-101

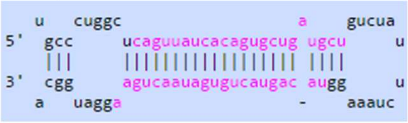

miR101-3p binding sites on SOX9: 7mer-m8 and 7mer-A1

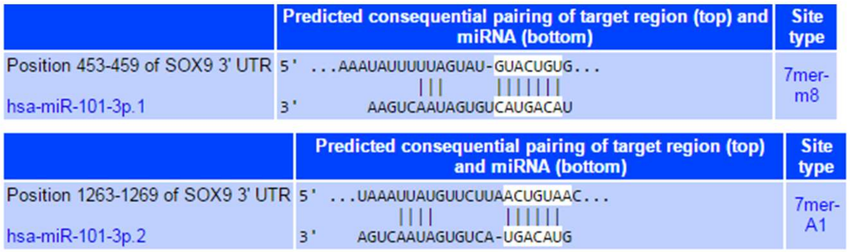

	Predicted consequential pairing of target region (top) and miRNA (bottom)	Site type
Position 453-459 of SOX9 3' UTR<br>hsa-miR-101-3p.1	5' ...AAAUAUUUUUAGUAU-GUACUGUG...<br>3' AAGUCAAUAGUGUCAUGACAU	7mer-m8

	Predicted consequential pairing of target region (top) and miRNA (bottom)	Site type
Position 1263-1269 of SOX9 3' UTR<br>hsa-miR-101-3p.2	5' ...UAAAUUAUGUUCUUAACUGUAAC...<br>3' AGUCAAUAGUGUCA-UGACAUG	7mer-A1

Figure S2. Predicted targets of miR-101-3P. A, Predicted miR101-3p binding sites on Cadherin 11 (CDH11): 7mer-m8. **B,** miR101-3p binding sites on SOX9: 7mer-m8 and 7mer-A1

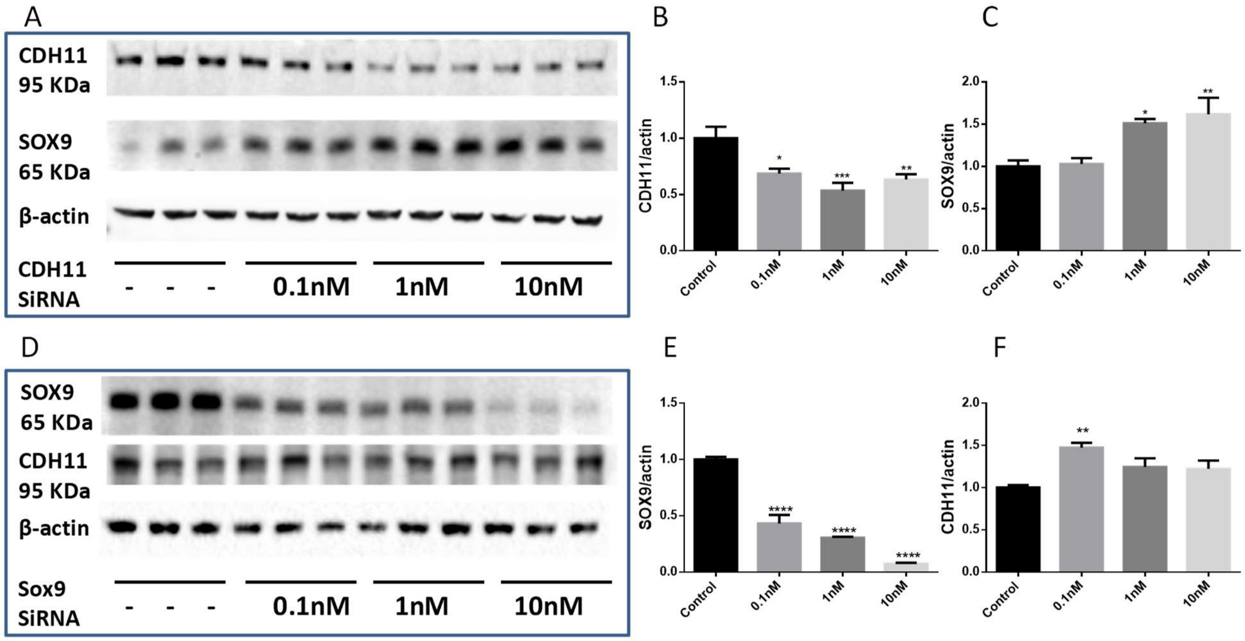

Figure S3. Cross regulation of CDH11 and SOX9 in HAVICs. A, Western blot analysis of CDH11 and SOX9 expression in HAVICs following transfection with CDH11-siRNA for 48 hours. **B,** Quantification of CDH11 expression. **C,** Quantification of SOX9 expression. **D,** Western blot analysis of Sox9 and CDH11 expression following transfection with Sox9-siRNA for 48 hours**. E,** Quantification of SOX9 expression. **F,** Quantification of CDH11 expression. Data=means±SEM. **p<0.01, ***p<0.001, ****p<0.0001 vs Control. N=3.

A.

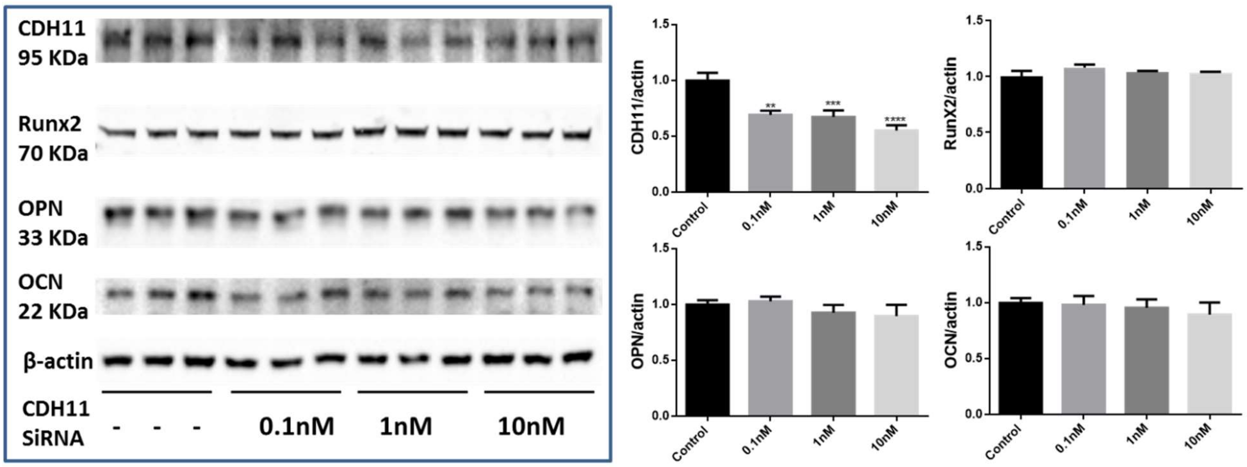

B.

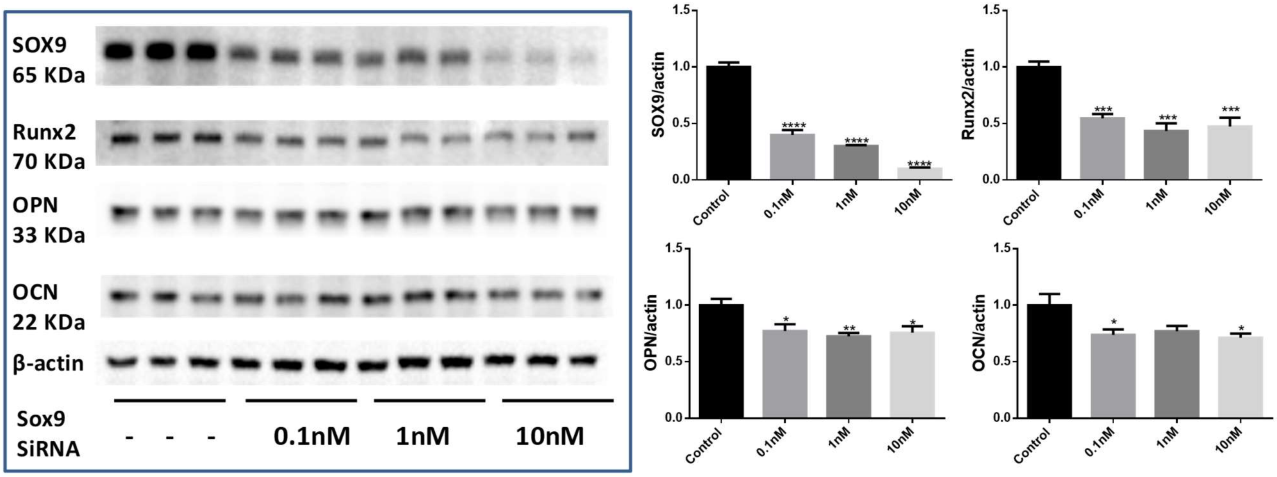

C.

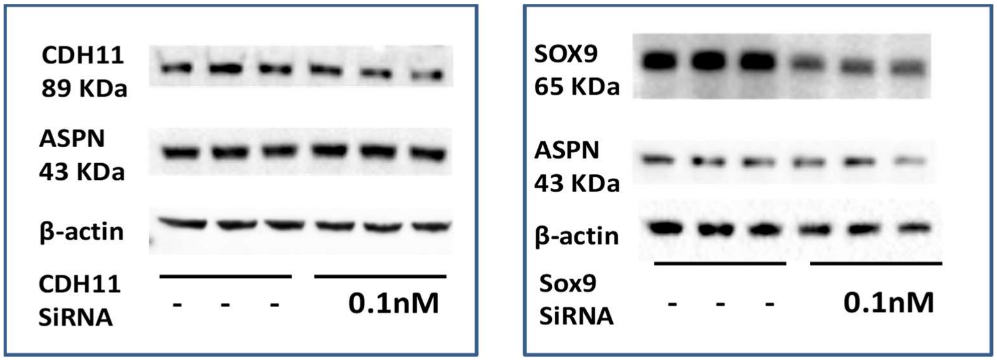

Figure S4. Knockdown of CDH11 or SOX9 alone was insufficient to induce osteogenesis. A, Western blot analysis of CDH11, Runx2, OPN, and OCN expression in

HAVICs following transfection with CDH11-siRNA for 48 hours. **B,** Western blot analysis of Sox9, Runx2, OPN, and OCN expression in HAVICs following transfection with SOX9-siRNA for 48 hours. **C,** Western blot analysis of CDH11, ASPN, and SOX9 following transfection with CDH11-shRNA or SOX9-shRNA for 48 hours. Data=means±SEM. **p<0.01, ***p<0.001, ****p<0.0001 vs Control. N=3.

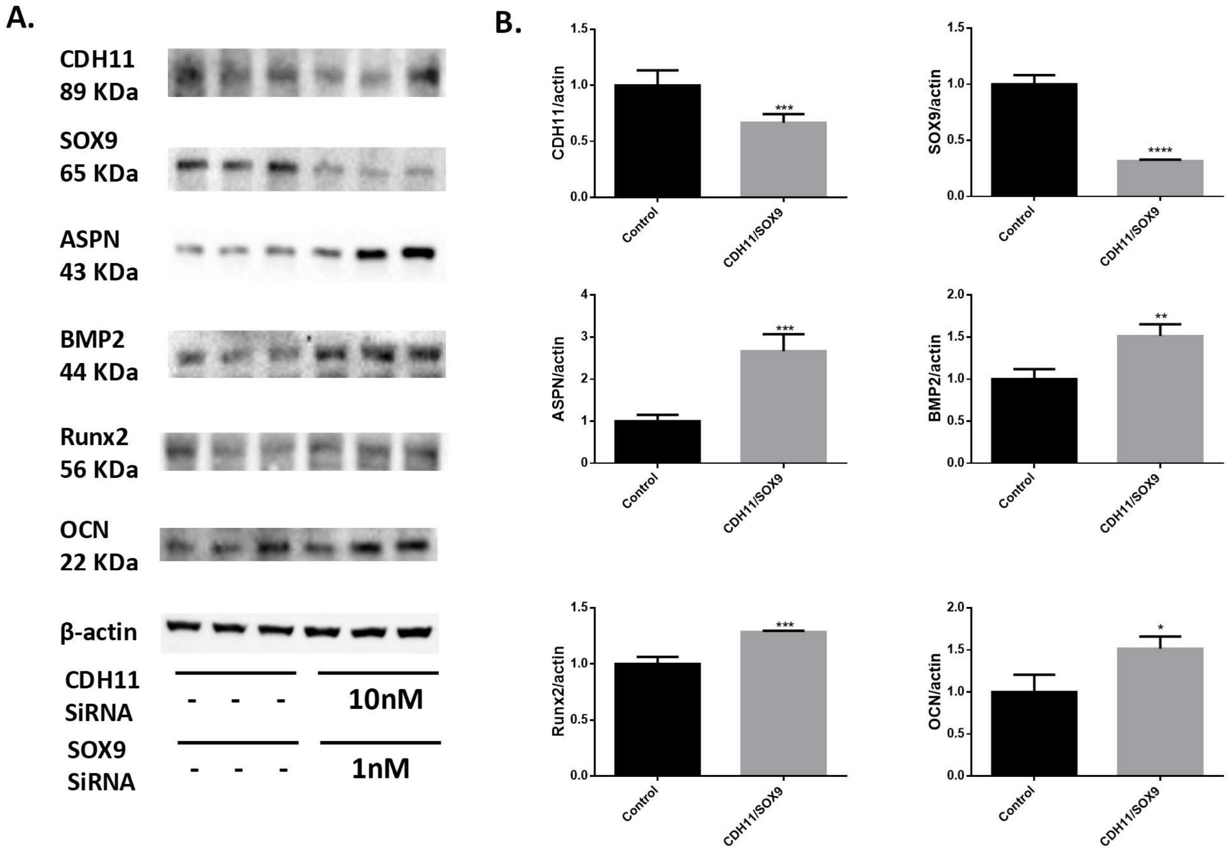

Figure S5. Knockdown of both CDH11 and SOX9 promoted osteogenesis in HAVICs. A, Western blot analysis of CDH11, SOX9, ASPN, BMP2, Runx2, and OCN expression in HAVICs following transfection with both CDH11-siRNA and SOX9-siRNA for 48 hours. **B,** Quantification of CDH11, SOX9, ASPN, BMP2, Runx2, and OCN expression in HAVICs. Data=means±SEM. **p<0.01, ***p<0.001, ****p<0.0001 vs Control. N=3.

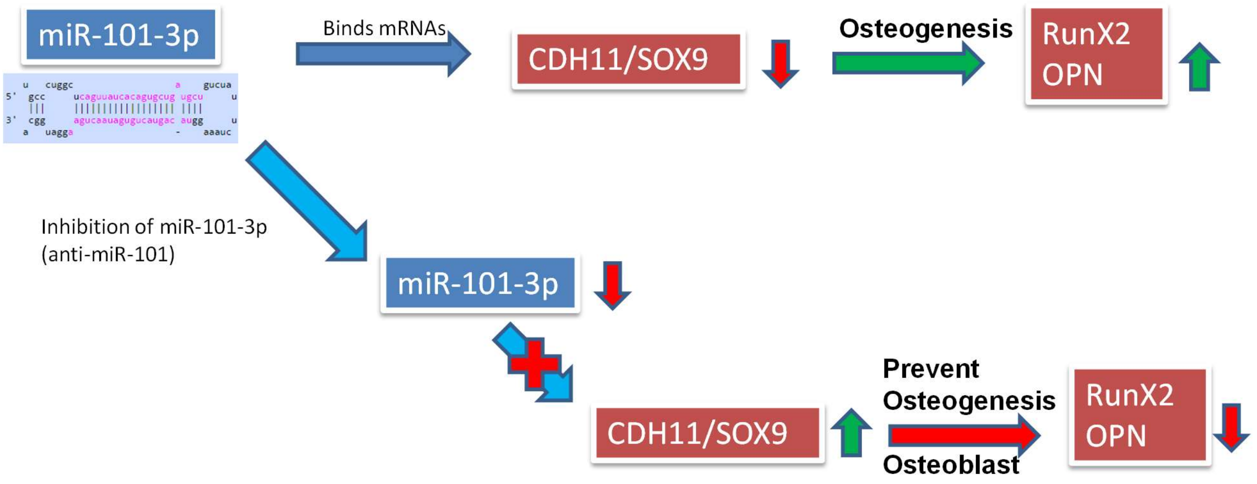

Figure S6. Central illustration of mechanistic pathways in regulating osteogenesis by miR-101-3p in human aortic valve interstitial cells (HAVICs).
